# General corruption scale: development and psychometric properties

**DOI:** 10.1186/s40359-026-04177-2

**Published:** 2026-02-16

**Authors:** João Gabriel Modesto, Ronaldo Pilati, Adriana Satico Ferraz, Mauricio Miranda Sarmet, Marília Mesquita Resende, Juliana B. Porto, Cícero Roberto Pereira

**Affiliations:** 1 State University of Goiás, Luziânia, Brazil; 2 Department of Work and Social Psychology, University of Brasília, Brasília, Brazil; 3 Belas Artes University Center of São Paulo, São Paulo, Brazil; 4 Federal Institute of Education, Science and Technology of Paraíba, João Pessoa, Brazil; 5 University Center of Brasília, Brasília, Brazil; 6 Institute of Social Sciences, University of Lisbon, Lisbon, Portugal

**Keywords:** Corruption, Dishonesty, Unethical behavior, Psychometrics

## Abstract

**Background:**

Corruption is a pervasive phenomenon that erodes institutional structures and undermines social relations. Despite its widespread impact, little attention has been given to developing and providing empirical evidence of the construct validity and reliability of measures assessing individual differences in support for corruption. This study seeks to address this gap by introducing, for the first time, the General Corruption Scale and presenting the results of a research program designed to provide robust empirical evidence for the quality of its psychometric properties.

**Methods:**

In the first study, content validity was assessed by three experts using the Content Validity Coefficient, confirming the adequacy of the scale’s content. Subsequent studies employed both exploratory (*n* = 308) and confirmatory (*n* = 840) factor analyses to evaluate its factorial structure.

**Results:**

Results supported a two-factor model comprising 26 items, organized into Perception of Corruption and Support for Corruption. The model demonstrated satisfactory fit indices and performed better than alternative measurement models. Evidence of nomological validity was established through associations with measures of *jeitinho* and propensity to trust.

**Conclusions:**

The scale is theoretically aligned with its foundational model and demonstrates evidence of validity and reliability. Further research is recommended to assess its psychometric performance across diverse contexts.

## Introduction

 Corruption is commonly defined as the abuse of entrusted power for private gain [1]. From a psychological research perspective, this definition entails inherent complexity, as the measurement of corruption requires research designs and instruments capable of capturing rule violations, abuses of power, and potential illicit gains. In a systematic review of instruments used to assess corruption in psychological research, Ponce-Díaz et al. [2] identified several limitations, including a lack of empirically supported content validity, unstable or unclear factorial structures, and insufficient evidence of convergent validity. The authors therefore emphasized the need for new studies aimed at developing and validating instruments that are theoretically grounded and supported by robust psychometric evidence.

In response to these recommendations, the present study introduces the General Corruption Scale (GCS), developed based on the Analytical Model of Corruption [3]. By incorporating conceptual complexity and multilevel predictors of corrupt behavior, this framework seeks to address key challenges in the measurement of corruption. Despite these methodological challenges, the literature offers several approaches to measuring corruption across disciplines [2, 4], particularly through widely used corruption indices. Judge et al. [5] identified three major indices frequently employed in corruption research: the *Control of Corruption Index* (CCI), the *Corruption Index* (CI), and the *Corruption Perceptions Index* (CPI).

The *Control of Corruption Index (*CCI*)*, developed by the World Bank as part of the Global Governance Indicators, assesses the perceived extent to which public power is exercised for private gain. Scores range from − 2.5 to + 2.5, with higher values indicating greater control over corruption. The index is constructed from multiple data sources, including assessments by non-governmental organizations and surveys conducted with private firms and public institutions [6, 7]. Similarly, the *Corruption Index (CI)*, produced by Political Risk Services (PRS), contributes to the Political Risk Index and ranges from 0 to 6, with higher scores reflecting lower levels of corruption. Since its introduction in 1980, this index has focused on dimensions such as the influence of business actors on political processes, the prevalence of bribery, and the extent to which clientelism supersedes merit-based practices [5]. Finally, the *Corruption Perceptions Index (CPI)*, compiled by Transparency International, measures perceived levels of public sector corruption based on global survey data. It ranks countries according to corruption levels and has become one of the most widely cited indicators in both academic and policy-oriented research. Despite its broad use and acceptable reliability, the CPI - like other perception-based measures - has been applied across multiple disciplines and contexts, often without being grounded in a clearly specified psychological measurement framework [8, 9].

When comparing these three major indices, Neumann and Graeff [10] found that the CI was less accurate in capturing the corruption construct. Both the CCI and the CPI exhibited limited discriminant validity, particularly in their ability to distinguish corruption from closely related concepts such as democracy. In addition, multicollinearity among these indices may undermine the accuracy and interpretability of results. More critically, none of these indices were developed based on theoretical frameworks that clearly define corruption or incorporate multilevel explanatory factors, nor were they supported by rigorous psychometric methodologies. Taken together, these limitations underscore the need for improved measurement tools, especially given the substantial influence these indices exert on public policy and global perceptions of corruption.

Whereas these indices primarily focus on aggregated societal indicators, micro-level assessments of corruption tend to examine individual-level dimensions such as corruption perception [11], tolerance toward corruption [12], and corruption intention [13, 14]. To identify micro-level measures within psychological research, Ponce-Díaz et al. [2] reviewed 24 studies and classified existing tools into three main categories: (a) self-report instruments, including scales, questionnaires, and inventories; (b) corruption-related scenarios and vignettes; and (c) experimental games involving bribery and corruption. This classification highlights both the diversity of approaches and the lack of standardized, theoretically grounded measures in the field.

Among self-report instruments, four notable studies specifically aimed to develop corruption-related measures. Guerrero-Martelo et al. [15], for example, validated a version of the *Defining Issues Test* focused on moral reasoning in corruption-related dilemmas. Despite its contributions, this instrument has limited scope, as its items are tied to specific scenarios and are not easily generalizable. Moreover, validation was conducted exclusively with university students in Colombia, and methodological limitations, such as insufficient clarity regarding factor extraction and rotation procedures, were noted [2].

In a related effort, Orellana and Bossio [16] developed the *Attitude Toward Corruption Scale* for university students. The final version comprises 20 items distributed across seven factors, derived through exploratory factor analysis with varimax rotation. Although the authors reported factor loading data, important limitations remain, including the absence of explicit sample selection criteria and missing sociodemographic information. Moreover, the scale’s applicability is restricted to populations that are unlikely to occupy positions of power, which limits its relevance for broader corruption research.

In a different approach, Lin et al. [17] introduced the *Multidimensional Scale of Employee Fraud Motive*, validated among working adults, a population more directly relevant to corruption-related phenomena. Their study was grounded in a theoretical model and provided strong evidence of validity. Nevertheless, the authors did not report detailed sociodemographic characteristics of the sample, nor did they offer a comprehensive description of participant selection. In addition, because the instrument was developed within the Chinese cultural context, its cross-cultural applicability should be tested.

Furthermore, Yang and Chen [18] created a 27-item scale assessing fraud - a corruption subtype - based on the Moral Disengagement Theory [19]. The authors conducted both exploratory and confirmatory factor analyses, presented evidence of factorial validity, and examined associations with personality traits. Although the study included company employees, it lacked detailed sociodemographic information. The authors also acknowledged that cultural specificities associated with the Chinese context may limit the broader applicability of their findings.

More recently, Ponce-Díaz et al. [14] presented the *Corruption Intention Scale*, developed based on the Theory of Planned Behavior (TPB) [20]. Starting from an initial pool of 47 items distributed across three TPB dimensions, namely perceived behavioral control, attitudes toward corruption, and subjective norms, the authors conducted two studies with Peruvian participants and proposed a final 12-item version. The scale demonstrated adequate psychometric indicators, including factorial and convergent validity. Although this effort represents an important theoretically guided contribution, the chosen theory is generic and focused on comprehending the relationship between attitudinal variables in social psychological literature. Consequently, the literature still lacks measurement efforts grounded in theoretical models specifically developed for social psychological research on corruption, particularly those that explicitly incorporate a multilevel perspective.

Although measurement efforts in the psychological literature have made considerable progress in assessing individual differences in corruption-related attitudes, several important gaps remain [2]. These gaps include sample-specific research designs, limited cross-cultural generalizability, the absence of a clearly articulated theoretical framework for defining corruption and its dimensions, and insufficient systematic empirical evidence addressing multiple forms of validity. Together, these limitations underscore the need for new instruments that are both theoretically grounded and psychometrically robust, and that can capture the complexity of corruption across diverse contexts.

In an effort to broaden the measurement of corruption processes by adopting a specific theoretical model, the present study contributes to advancing corruption measurement in the psychological literature in three main ways. First, it offers an operational definition of corruption grounded in a theoretically explicit framework, namely the Analytical Model of Corruption [3], which provides more nuanced theoretical guidance than generic models of social behavior. Second, it addresses previously identified limitations by integrating theoretical coherence and psychometric rigor throughout the scale development process. Third, it proposes a scale intended to be applicable to diverse populations and contexts, thereby enhancing its potential for cross-cultural use and its relevance for individuals who may occupy positions of power.

### The analytical model of corruption

The present measure was developed based on the *Analytical Model of Corruption* (AMC) [3], in line with the recommendation by Ponce-Díaz et al. [2] regarding the importance of employing well-defined theoretical frameworks in scale validation studies on corruption. Within this model, corruption is defined as the abuse of power for private gain. The AMC adopts a multilevel perspective, proposing that corruption should be examined through the interaction of micro, meso, and macro level factors, as well as a positional dimension.

At the micro level, the model focuses on individual-level factors such as personality traits [21] and cognitive decision-making processes, which include both rational and deliberative mechanisms [11, 22] as well as more automatic or intuitive processes [23, 24]. The meso level addresses group dynamics, including in-group favoritism [25] and the role of social identities in shaping corrupt behavior [26]. At the macro level, the model incorporates broader contextual influences, particularly cultural factors, recognizing how social norms and institutional environments contribute to the prevalence and perception of corruption [27]. In addition, the AMC introduces a positional dimension that refers to the individual’s role or hierarchical status within a corrupt situation. This dimension represents a key contribution of the model, as it highlights how positions of power shape both the likelihood of engaging in corrupt acts and the moral evaluation of such behavior.

### Study overview

The development and psychometric evaluation of the *General Corruption Scale* (GCS) were conducted across three studies. Study 1 focused on item development and the assessment of content validity. Study 2 aimed to provide initial empirical evidence of factor validity based on the internal structure of the scale, using exploratory procedures to examine the data, identify the latent factors underlying the corruption construct, and assess internal consistency. Study 3 further advanced the validation process by providing confirmatory evidence of factor validity through confirmatory factor analysis (CFA), estimating parameters of the internal structure, and comparing the previously identified factor solution with alternative models. In addition, this study sought to establish evidence of nomological validity, thereby strengthening the theoretical and empirical foundations of the scale.

## Study 1

### Method

#### Participants

Three expert judges participated in the study, all holding PhDs in Psychology and having experience developing psychometric instruments. On average, these experts had 4.33 years of experience (SD = 3.09) conducting research in corruption-related areas.

### Research procedures

#### Construction of the general corruption scale

Based on the Analytical Model of Corruption (AMC), the proposed measure was designed to be assessed primarily at the micro level of analysis, as it focuses on individuals’ evaluations and judgments regarding corruption. Item development was conducted by two researchers who considered common corruption-related situations within the Brazilian context to ensure conceptual relevance and clarity. The constitutive and operational definitions guiding the construction of the scale were grounded in the assumption that micro-level corruption comprises three distinct yet interrelated dimensions: (1) corruption perception, defined as the individual’s evaluation of how prevalent corruption is in a given context and the likelihood that others would engage in it [11], with higher scores indicating a stronger perception that corruption is likely and frequent, rather than a value judgment about whether the phenomenon is positive or negative; (2) tolerance of corruption, understood as the degree to which corrupt practices are considered acceptable or justifiable [12], with higher scores reflecting a greater tendency to view such practices as legitimate or understandable; and (3) corruption intention, a self-referential evaluation indicating the individual’s willingness to engage in corrupt behavior [13], with higher scores representing a greater personal disposition to act in this manner.

In line with the AMC’s multilevel perspective, the item development process incorporated elements from all analytical levels. For example, several items describe corruption scenarios involving specific social categories, such as Brazilians, politicians, and public servants, which correspond to group-based processes characteristic of the meso level. In addition, macro-level cultural aspects were incorporated into the item content, including the cultural syndrome of Brazilian *jeitinho*, understood as a socially accepted strategy for navigating complex or restrictive systems [28, 29]. These elements were included to reflect broader contextual influences on corrupt behavior. The positional dimension was also addressed, with certain items capturing differences between more active and more passive roles within corruption scenarios. The initial version of the scale consisted of 27 items, as presented in Table 1. Responses were recorded using a Likert-type scale, with response formats and labeling designed to ensure clarity and ease of interpretation.

**Table 1:** Original scale items

Perception
1 - Brazilians attempt to offer a reward to a public servant to speed up bureaucratic procedures.
2 - Politicians attempt to receive a reward for the benefits they provide to the population.
3 - Public servants expect a reward to speed up bureaucratic procedures.
4 - Brazilians are willing to offer a gift as a small token of appreciation to a public servant who resolved an issue for them.
5 - Politicians try to receive their share in exchange for speeding up the approval of projects that benefit the population.
6 - Public servants are willing to accept a gift as a small token of appreciation for their work.
7 - Brazilians attempt to return the favor a public servant did by resolving bureaucratic matters.
8 - Politicians seek power as a means to obtain personal benefits.
9 - Public servants are willing to accept a favor in return for resolving a bureaucratic issue.
Tolerance
10 - It is acceptable to offer some reward to a public servant to speed up bureaucracy.
11 - It is natural for a politician to receive a reward for the benefits they provide to the population.
12 - It is understandable that public servants expect a reward to speed up bureaucratic procedures.
13 - It is acceptable to offer a gift as a small token of appreciation to a public servant who resolved an issue for us.
14 - It is understandable for a politician to receive their share in exchange for speeding up the approval of projects that benefit the population.
15 - It is natural for public servants to be willing to accept a gift as a small token of appreciation for their work.
16 - It is good to return the favor a public servant did for us by resolving bureaucratic matters.
17 - It is understandable that politicians seek power as a means to obtain personal benefits.
18 - It is acceptable for public servants to be willing to accept a favor in return for resolving a bureaucratic issue.
Intention
19 - I would be willing to offer a reward to a public servant to speed up bureaucratic procedures.
20 - If I were a politician, I would seek to receive a reward for the benefits I provided to the population.
21 - If I were a public servant, I would expect to be offered a reward for speeding up a bureaucratic procedure.
22 - I would be willing to offer a gift as a small token of appreciation to a public servant who resolved an issue for me.
23 - If I were a politician, I would seek to receive my share in exchange for speeding up the approval of projects that benefited the population.
24 - If I were a public servant, I would be willing to accept a gift as a small token of appreciation for my work.
25 - I would try to return the favor a public servant did for me by resolving bureaucratic matters.
26 - If I were a politician, I would seek power as a means to obtain personal benefits.
27 - If I were a public servant, I would be willing to accept a favor in return for resolving a bureaucratic issue.

#### Expert judges protocol

The evaluation protocol was developed based on the Content Validity Coefficient (CVC) framework, in accordance with APA guidelines for gathering evidence of content validity [30]. Four criteria were assessed: clarity of language (CL), practical relevance (PR), theoretical relevance (TR), and theoretical dimension (TD). For the CL, PR, and TR criteria, items were rated on a five-point scale ranging from 1 (not at all appropriate) to 5 (extremely appropriate). For the TD criterion, judges were asked to classify each item according to its alignment with one of the theoretical dimensions of the General Corruption Scale: Perception, Intention, or Tolerance. The final section of the protocol included an open-ended question that allowed judges to provide comments and suggestions for improving the items.

### Data collection and analysis procedures

Judges were selected based on their expertise in corruption research. The link to the Judge Evaluation Protocol was distributed via email and a messaging application. The evaluation was conducted remotely and asynchronously using the Google Forms platform. Prior to participating in the content validity evaluation, all expert judges received an informed consent statement electronically. The statement described the purpose of the study, the procedures involved in evaluating the scale items, and the voluntary nature of participation. Judges were informed that their responses would be treated confidentially, that no identifying information would be collected, and that they could decline participation or withdraw at any time without any consequences. Only experts who provided explicit consent proceeded to complete the evaluation protocol.

The Content Validity Coefficient (CVC) for the criteria of clarity of language (CL), practical relevance (PR), and theoretical relevance (TR) was calculated using an Excel spreadsheet, following the recommendations of Cassepp-Borges et al. [31]. CVC values equal to or greater than 0.80 were considered indicative of item adequacy. For the theoretical dimension (TD) criterion, Fleiss’ Kappa (k) was computed to assess inter-rater agreement. The interpretation of k values followed conventional benchmarks, with values between 0.40 and 0.60 indicating fair agreement, values between 0.61 and 0.75 indicating good agreement, and values above 0.75 indicating excellent agreement. The free-marginal Kappa method was adopted because raters were not required to assign a fixed number of items to each theoretical dimension, which is consistent with the approach recommended by Brennan and Prediger [32]. To analyze the open-ended responses, a deductive thematic analysis was conducted using a reliability coding approach. As recommended by Souza [33], this method is appropriate for evaluating content validation data derived from expert feedback.

## Results

Table 2 presents the results of the Content Validity Coefficient (CVC) calculations for the criteria of clarity of language (CL), practical relevance (PR), and theoretical relevance (TR), as well as the Fleiss’ Kappa (k) values related to the assessment of the theoretical dimension (TD). These results correspond to the items and respective factors of the *General Corruption Scale*, reflecting the content analysis conducted to evaluate the scale’s theoretical structure.

**Table 2:** Content Validity Coefficients for the Items and Factors of the General Corruption Scale

**Perception Factor**	**Final CVC of items**			**CVC factor**
	**CL**	**PR**	**TR**	***k***
Item 1	.763	.829	.829	1
Item 2	.896	.629	.629	1
Item 3	.829	.829	.829	1
Item 4	.829	.829	.829	1
Item 5	.629	.829	.829	1
Item 6	.829	.829	.829	1
Item 7	.696	.829	.829	1
Item 8	.963	.963	.963	1
Item 9	.829	.963	.963	1
Total	.807	.837	.837	1

**Tolerance**	**Final CVC of items**			**CVC factor**
	**CL**	**PR**	**TR**	***k***
Item 10	.963	.963	.963	1
Item 11	.896	.696	.696	1
Item 12	.829	.896	.896	1
Item 13	.829	.829	.829	1
Item 14	.896	.963	.963	1
Item 15	.829	.829	.829	1
Item 16	.829	.829	.829	1
Item 17	.963	.963	.963	1
Item 18	.896	.896	.896	1
Total	.881	.874	.874	1

**Intention Factor**	**Final CVC of items**			**CVC factor**
	**CL**	**PR**	**TR**	***k***
Item 19	.958	1	.963	1
Item 20	.950	.800	.763	1
Item 21	.950	1	.963	1
Item 22	.925	.866	.829	1
Item 23	.925	1	.963	1
Item 24	.851	.866	.829	1
Item 25	.851	.866	.829	1
Item 26	.629	1	.963	1
Item 27	.629	1	.963	1
Total	.852	.896	.896	1

*CL* Clarity of language, *PR* Practical relevance, *TR* Theoretical relevance

Table 2 indicates that Items 5 and 7 of the Perception factor, as well as Items 26 and 27 of the Intention factor, presented CVC values below 0.80 for the clarity of language (CL) criterion. Regarding practical relevance (PR) and theoretical relevance (TR), Item 2 of the Perception factor and Item 11 of the Tolerance factor were classified as unsatisfactory. Overall, 92.59% of the CVC values met the adequacy criteria. This result is further supported by the qualitative analysis of the experts’ comments, which did not identify any structural issues that would justify item reformulation or exclusion. Accordingly, the General Corruption Scale retained its original structure comprising 27 items.

## Study 2

This study aimed to provide evidence of validity based on the internal structure of the scale by examining the underlying factorial structure of the corruption construct through exploratory factor analysis.

### Method

#### Participants

Following the general guideline of at least 10 participants per item [34], the target sample size was set at 270 participants. The final sample comprised 308 Brazilian participants, aged between 18 and 75 years (M = 38.04, SD = 14.16). Of the total sample, 61% identified as women and 39% as men. Participants were drawn from all five regions of Brazil, with 50.3% from the Southeast, 18.5% from the Northeast, 16.6% from the South, 9.1% from the Central-West, and 5.5% from the North. Socioeconomic status was assessed using national classification criteria [35], yielding the following distribution: 13.6% in Class A, 13.3% in Class B1, 24.4% in Class B2, 22.7% in Class C1, 17.2% in Class C2, and 8.8% in Class DE. According to this classification system, Class A corresponds to higher income levels, Classes B and C represent middle income levels, and Class DE represents lower income levels.

### Measure

The same version of the *General Corruption Scale* used in Study 1 was applied to assess corruption. The measure was administered as a self-report questionnaire. Participants were instructed to indicate their level of agreement with each statement using a five-point Likert-type scale ranging from 1 (strongly disagree) to 5 (strongly agree). The initial version of the scale was theoretically conceived to assess three dimensions of corruption, namely *Perception of Corruption*, *Tolerance toward Corruption*, and *Corruption Intention*. Scores for each dimension were computed by calculating the mean of the items corresponding to each factor. In addition, a sociodemographic questionnaire was administered to collect information about participants’ profile.

### Procedures of data collection and analysis

To obtain a sample nearly representative of the Brazilian population, data collection was outsourced to a company specializing in online research (https://www.offerwise.com/?lang=en). The sampling procedure involved inviting participants from all Brazilian states and regions, based on a pre-registered group of participants encompassing all socio-economic strata of the Brazilian population. An attention check item was included in the questionnaire: “As a way to check your attention to the questionnaire, mark two as the answer to this question.” Participants who failed this item were excluded, reducing the initial sample from 638 to 308 respondents. It is evident that the reduction in the final number of participants, combined with self-selection into the study, resulted in a sample that cannot be considered representative of the Brazilian population. Nevertheless, the final sample was sufficiently diverse from a demographic perspective to allow an appropriate examination of the scale’s factorial structure. Before completing the online questionnaire, participants were presented with an informed consent form describing the study’s general purpose, the voluntary nature of participation, and the procedures involved. The consent form explicitly stated that participation was anonymous, that no personally identifiable information would be collected, and that all responses would be kept confidential and used exclusively for research purposes. Participants were informed of their right to discontinue participation at any time without penalty, and only those who actively agreed to the informed consent statement were allowed to access and complete the survey.

Before conducting the Exploratory Factor Analysis (EFA), assumptions of normality and factorability of the dataset were assessed. The number of factors to be extracted was determined using the Hull method [36]. Once the appropriate number of factors was identified, the factor solution was examined by comparing rotated and unrotated solutions. Based on this comparison, the distribution of items across factors was evaluated, and items with insufficient factor loadings or cross-loadings across multiple factors were considered for exclusion. Items were retained if they presented a primary factor loading ≥ 0.30 and cross-loadings ≤ 0.20, with a minimum difference of 0.20 between the primary loading and any secondary loading. The Root Mean Square Residual (RMSR) was used as a fit index to assess the model’s plausibility. All analyses were conducted using the Factor software. Reliability was assessed using Cronbach’s alpha and the Omega coefficient.

## Results

The correlation matrix was examined to assess the adequacy of the dataset for Exploratory Factor Analysis and met the criteria for factorability (Bartlett’s test of sphericity = 2684.80, df = 351, *p* <.001; KMO = 0.83). Tests of multivariate normality indicated deviations from normality in kurtosis, as revealed by Mardia’s test (z = 36.40, *p* <.001). Based on the Hull method, a two-factor solution was identified as presenting the best fit indices, as shown in Table 3.

**Table 3:** Adequacy indices by number of factors according to the hull method

Number of factors	Goodness of fit	DF	Scree test
0	0.000	351	0.000
1	0.693	324	2,604
2	0.949	298	4,941*
3	0.999	273	0.000
4	0.999	249	

* Most recommended alternative

The two-factor structural solution was selected for the factor analysis, yielding an RMSR value of 0.072. To examine the distribution of items based on their factor loadings, a factor analysis with promin rotation was conducted. The resulting item distribution across factors is presented in Table 4.

**Table 4:** Factor loadings and item distribution for each factor of the general corruption scale

Item	Factor 1	Factor 2
1	**0.683**	− 0.220
2	**0.569**	− 0.211
3	**0.541**	− 0.052
4	**0.656**	− 0.018
5	**0.636**	− 0.128
6	**0.613**	− 0.073
7	**0.460**	0.051
8	**0.541**	− 0.125
9	**0.466**	− 0.147
10	0.154	0.194
11	− 0.081	**0.406**
12	− 0.117	**0.389**
13	0.002	**0.420**
14	− 0.019	**0.531**
15	0.098	**0.505**
16	0.121	**0.533**
17	0.138	**0.521**
18	− 0.030	**0.596**
19	0.218	**0.498**
20	0.007	**0.614**
21	0.096	**0.595**
22	0.062	**0.522**
23	0.011	**0.580**
24	0.043	**0.565**
25	0.035	**0.577**
26	− 0.026	**0.462**
27	0.089	**0.530**
Internal consistency	**α = 0.85** **ω = 0.86**	**α = 0.93** **ω = 0.93**

The values in bold represent the factor to which the item was associated

Only one item (item 10 – “It is acceptable to offer some reward to a public servant to speed up bureaucracy”) failed to achieve an acceptable factor loading (i.e., greater than 0.30) on any factor. However, initially, item 10 was retained for subsequent statistical analyses aimed at examining the instrument’s internal structure. The item content represents a socially embedded set of attitudinal and cultural characteristics of the *Brazilian jeitinho*, manifested in practices involving the offering of advantages to expedite bureaucratic procedures [28, 29].

Content analysis of the remaining items allowed for the conceptual definition of the two resulting factors. Factor 1, labelled *Perception of Corruption*, includes items related to how corruption is perceived by Brazilians. Factor 2, labelled *Support for Corruption*, includes items reflecting the respondent’s acceptance of corrupt practices and intention to engage in such behaviors. The internal consistency of both factors was satisfactory, and the factors showed a weak positive correlation (*r* =.12, *p* =.034). To further evaluate the factorial and nomological validity of the scale, additional evidence was gathered in Study 3, which involved confirmatory factor analysis using a new dataset, and nomological validity to assess the theoretical consistency of the measure.

## Study 3

In Study 3, using an independent sample, we conducted confirmatory factor analyses to evaluate the two-factor structure identified in Study 2 against two alternative models: (a) a single-factor model representing a generalized attitude toward corruption and (b) the initially hypothesized three-factor model comprising perception, tolerance, and intention. This study also sought to examine evidence of nomological validity of the *General Corruption Scale* by assessing its associations with measures of *jeitinho* [37] and propensity to trust [38, 39].

*Jeitinho* is a well-studied Brazilian cultural syndrome, defined as a mechanism for navigating complex contexts that involves creativity, efforts to preserve a positive social atmosphere, and, at times, the violation of laws and social norms to achieve goals [28, 29, 40–43]. The construct is organized into two dimensions: *Jeitinho Simpático*, which involves creative problem solving and the cultivation of positive interpersonal relationships, and *Jeitinho Malandro*, which involves behavioral strategies that include norm breaking to achieve personal goals [37]. It was hypothesized that *Jeitinho Malandro* would be positively associated with corruption, thereby providing evidence of nomological validity. For example, previous research has linked *jeitinho* to unethical behavior in the workplace [44]. Propensity to trust, defined as the general tendency to believe that others are trustworthy [45], was also included in the assessment of nomological validity. In this case, higher levels of distrust were expected to be positively associated with corruption scores, based on previous research indicating that distrust is a predictor of corruption [46].

### Method

#### Participants

For the test of nomological validity using two additional measures, a power analysis indicated that, assuming an expected correlation effect size of *r* =.10, an alpha level of 0.05, and a statistical power of 0.95, a minimum sample size of 1,200 participants would be required. After excluding responses that failed the attention check, the final analytic sample comprised 840 Brazilian participants, of whom 50.7% identified as women and 49.3% as men. Regarding age distribution, 15.7% of participants were between 18 and 24 years old, 31.5% between 25 and 34, 24.2% between 35 and 44, 21.9% between 45 and 59, 4.5% between 60 and 64, 1.9% between 65 and 74, and 0.2% were 75 years old or older. Socioeconomic status was assessed according to national classification criteria [35], with 14.8% of participants classified as Class A, 39.5% as Class B, 37.3% as Class C, and 9.5% as Class DE. Participants represented all five geographic regions of Brazil: Southeast (43.3%), South (17.3%), Central-West (18.0%), Northeast (11.0%), and North (10.5%). After excluding participants who failed the attention check, the observed statistical power for detecting the estimated correlation effects was 0.83.

### Instruments

#### General corruption scale

The original version of the General Corruption Scale, consisting of 27 items, was used as described in Study 1 and 2.

#### Personal jeitinho scale

*The Personal Jeitinho Scale* [37] comprised 10 items (e.g. She/He get in a party without fee because she/he knows the party productor). The original two-factor structure was replicated in the present study. The first factor, *Jeitinho Simpático*, comprised five items, with factor loadings ranging from 0.424 to 0.740 and demonstrated satisfactory reliability (α = 0.73; ω = 0.74). The second factor, *Jeitinho Malandro*, also included five items, with loadings ranging from 0.433 to 0.639, and showed acceptable reliability (α = 0.62; ω = 0.65).

#### Propensity to trust scale

Ten items (e.g. I trust others) from previously validated scales [38, 39] were used. In the present study, the items were organized into two factors: *Trust in Others* (factor loadings ranging from 0.455 to 0.833) and *Distrust* (loadings ranging from 0.316 to 0.933). Both factors demonstrated satisfactory internal consistency (Trust in Others: α = 0.86; ω = 0.86; Distrust: α = 0.73; ω = 0.75).

### Data collection and analysis procedures

As in Study 2, data collection was conducted by a specialized company (https://www.offerwise.com/?lang=en) through an online survey. Initially, 1.279 participants responded to the questionnaire. However, 439 participants were excluded for failing to answer the attention check item correctly. Although this substantial exclusion of participants reduced the initially planned statistical power, it was necessary to improve the overall quality of the data by retaining only respondents who demonstrated adequate attention to the questionnaire. Online survey studies pose several methodological challenges, particularly with respect to inattentive responding. In this context, we adopted best practices regarding attention checks to ensure that only participants who were attentive to the task were included in the final analysis. As in Study 2, participants in Study 3 were required to provide informed consent prior to data collection. At the beginning of the online survey, participants were presented with an informed consent statement describing the objectives of the study, the voluntary nature of participation, and the confidentiality of the data. The statement emphasized that responses were anonymous, that no identifying information would be collected, and that participants could withdraw from the study at any time without any negative consequences. Access to the questionnaire was granted only after participants explicitly indicated their consent.

Given the identified deviations from normality and the ordinal nature of the response scale, the Diagonally Weighted Least Squares (DWLS) estimation method was used to perform the Confirmatory Factor Analysis (CFA). Fit indices were generated for three competing models: a single-factor model, the two-factor model suggested by the exploratory factor analysis in Study 2, and the original three-factor model proposed during the scale’s development. These models were compared to the null model and one another, allowing for selecting the structure with the best fit.

Model comparisons were based on the following fit criteria: Comparative Fit Index (CFI) ≥ 0.90; Goodness of Fit Index (GFI) ≥ 0.90; Root Mean Square Error of Approximation (RMSEA) ≤ 0.08; and Standardised Root Mean Square Residual (RMSR) ≤ 0.08 [47]. All analyses were conducted using R software, employing the *lavaan* package.

## Results

### Confirmatory factor analysis

The models tested in the Confirmatory Factor Analysis (CFA) were compared to a null model. As shown in Table 5, all models were found to be statistically distinct from the null model, indicating that each represents a potentially viable solution. Subsequently, fit indices were analyzed to determine which model best represents the factorial structure of the scale.

**Table 5:** Fit statistics for Single-, Two-, and Three-Factor models

Models	Χ²	df	*p*	GFI	CFI	TLI	RMSEA	SRMR
1-factor	15114.448	324	< 0.001	0.878	0.597	0.564	0.233 [0.230; 0.236]	0.254
2-factor	1890.936	322	< 0.001	0.982	0.957	0.954	0.076 [0.073; 0.079]	0.090
3-factor	2728.813	321	< 0.001	0.985	0.934	0.928	0.095 [0.091; 0.098]	0.082

As shown in Table 5, the two-factor model demonstrated the best overall fit compared to the alternatives, except for the SRMR index, which slightly exceeded the recommended threshold of 0.08. Nevertheless, considering the favorable values of the other fit indices and the structure suggested by Study 2, the two-factor solution was retained as the most appropriate representation of the General Corruption Scale. Table 6 shows that all items demonstrated adequate factor loadings, indicating their relevance within the scale structure.

**Table 6:** Factor loadings of the items of the general corruption Scale, according to the Two-Factor model

Factor						95% CI	
	Item	Load	Standard error	*Z*	*p<*	LB	UB
Perception of Corruption	1	0.692	0.016	43,282	0.001	0.661	0.724
	2	0.818	0.014	57,890	0.001	0.790	0.845
	3	0.814	0.011	75,402	0.001	0.793	0.835
	4	0.701	0.015	46,522	0.001	0.672	0.731
	5	0.781	0.014	57,140	0.001	0.754	0.808
	6	0.823	0.010	79,039	0.001	0.802	0.843
	7	0.712	0.014	49,742	0.001	0.684	0.740
	8	0.732	0.017	42,846	0.001	0.699	0.766
	9	0.824	0.010	79.108	0.001	0.803	0.844
Support for Corruption	10	0.779	0.016	47,390	0.001	0.747	0.812
	11	0.729	0.019	37,505	0.001	0.691	0.767
	12	0.765	0.017	43,991	0.001	0.731	0.799
	13	0.776	0.015	50,710	0.001	0.746	0.806
	14	0.814	0.017	49,271	0.001	0.782	0.846
	15	0.748	0.017	44,474	0.001	0.715	0.781
	16	0.794	0.014	54,967	0.001	0.766	0.823
	17	0.721	0.020	35,851	0.001	0.681	0.760
	18	0.820	0.015	55.198	0.001	0.791	0.849
	19	0.778	0.016	48,830	0.001	0.747	0.809
	20	0.861	0.013	65,727	0.001	0.836	0.887
	21	0.865	0.013	68,837	0.001	0.840	0.890
	22	0.795	0.015	53,236	0.001	0.766	0.824
	23	0.825	0.015	55,493	0.001	0.796	0.854
	24	0.787	0.015	52,689	0.001	0.758	0.817
	25	0.791	0.015	54,387	0.001	0.762	0.819
	26	0.827	0.015	56,718	0.001	0.798	0.856
	27	0.833	0.014	58,934	0.001	0.805	0.861

*LB* Lower bound of the confidence interval, *UB* Upper bound of the confidence interval

Despite the adequacy of item 10 in the confirmatory factor analysis and considering the findings of the exploratory factor analysis conducted in Study 2, we propose its removal from the final version of the General Corruption Scale, as items 12 and 18 likewise assess the moral and attitudinal acceptance of undue instrumental interactions within the context of public bureaucracy, thus constituting substantially equivalent content [1, 28].

### Evidence of Nomological validity

Based on the final version of the scale, the *General Corruption Scale* was tested for nomological validity using the *Jeitinho* and Propensity to Trust measures, as shown in Table 7. As expected, the GCS dimensions were positively associated with *Jeitinho Malandro* and Distrust. The association between Support for Corruption and *Jeitinho Malandro* was moderate in magnitude, whereas the remaining correlations were small, indicating weak but theoretically meaningful relationships. Together, these findings support the nomological validity of the GCS.

**Table 7:** Pearson Correlation Matrix with Nomological Validation Evidence

	**Perception**	**Support**	**JS**	**JM**	**Trust**
Perception	—				
Support	.105**	—			
JS	.139***	-.087*	—		
JM	.071*	.396***	-.204***	—	
Trust	-.044	-.014	.294***	-.060	—
Distrust	.123***	.138***	-.051	.161***	-.411***

*JS(Jeitinho Simpático*)*,* *JM(Jeitinho Malandro)*

* p <.05, ** p <.01, *** p <.001

### General discussion

This research program details the development process of the GCS and provides, for the first time, robust empirical evidence supporting the content, factorial, and nomological validity of its scores, thereby establishing a solid foundation for its use in future psychological and cross-cultural research. The development of this instrument represents an important advancement in the field, as it is explicitly grounded in a theoretical model of corruption that differs from most existing measures. Unlike previous tools, which often lack specificity for capturing the socio-psychological processes underlying corruption, the GCS directly addresses these gaps. In doing so, it responds to the limitations identified by Ponce-Díaz et al. [2] by offering a theoretically coherent and psychometrically rigorous framework for assessing individual differences in corruption-related attitudes. This contribution is particularly relevant given the understanding that corruption is not only an institutional phenomenon, but also a psychological and social process shaped by individual evaluations, social norms, and contextual cues [3].

In Study 1, the construction of the General Corruption Scale (GCS) involved the development of items grounded in the Analytical Model of Corruption [3], ensuring strong theoretical alignment. The original version of the scale was evaluated by expert judges, who assessed item clarity, relevance, and theoretical consistency. Thus, the evidence of content validity reflects direct correspondence between the theoretical assumptions of the AMC and the operationalization of corruption in the scale items. This model incorporates the positional dimension, defined as the individual’s role or power position in a given situation, as well as macro level contextual elements, meso level social categories, and micro level self-referential evaluations of corrupt behavior. By integrating these analytical levels into item content, the GCS moves beyond narrowly defined corruption scenarios and allows for a more comprehensive representation of how individuals cognitively and normatively evaluate corruption across social roles and contexts. This theoretical grounding represents a meaningful step forward in the development of corruption measures anchored in a more nuanced understanding of the phenomenon, with implications not only for research but also for applied discussions on corruption prevention and public policy design.

In Studies 2 and 3, diversified samples from the Brazilian population were used, which represents improvement over prior research that relied on student samples or failed to report sampling criteria and participant characteristics [2]. The exploratory factor analysis conducted in Study 2 suggested a two-factor structure. In this solution, the *Perception of Corruption* dimension [11], which captures evaluations of corruption prevalence and the perceived likelihood of others engaging in corrupt behavior, retained its original items. In contrast, the dimensions of tolerance toward corruption [12] and corruption intention [13] converged into a single factor. This empirical convergence suggests that, at the individual level, judgments about the acceptability of corruption and one’s willingness to engage in corrupt behavior may reflect a shared underlying attitudinal orientation toward supporting corruption. Rather than representing distinct psychological processes, tolerance and intention appear to overlap substantially in how individuals evaluate and justify corrupt practices, which provides a plausible explanation for their empirical integration.

As a result, the final two-factor structure comprised *Perception of Corruption* and *Support for Corruption*. Notably, this organization preserves a theoretically meaningful distinction between evaluations of others’ behavior and self-referential orientations toward corruption, a distinction that has also been observed in previous research [11, 13]. This differentiation is consistent with broader findings in social psychology indicating that individuals often apply different normative standards when evaluating their own potential behavior versus the behavior of others. Only one item, Item 10, failed to load above the established threshold and was proposed for exclusion based on its empirical performance.

In Study 3, confirmatory factor analysis supported the two-factor structure identified in Study 2. Although Item 10 did not adversely affect model fit indices, its exclusion did not compromise the overall factorial structure, reinforcing the robustness of the remaining items. As a result, the final version of the General Corruption Scale comprised 26 items. The factor loadings observed in the confirmatory model indicate that the retained items contributed consistently and substantially to their respective dimensions, supporting the internal coherence of the scale and the adequacy of its measurement properties.

The resulting measure was also tested for nomological validity. As expected, scores on the GCS were positively associated with the *Jeitinho Malandro* dimension [37] and with distrust in others [46]. The moderate association between Support for Corruption and *Jeitinho Malandro* is theoretically meaningful, as both constructs involve a propensity to violate social norms for personal benefit [37]. In contrast, the smaller associations observed with distrust and with the perception dimension suggest that corruption-related attitudes are embedded in a broader network of psychological dispositions, each contributing incrementally rather than redundantly to the construct. Together, these findings provide initial but theoretically coherent support for the nomological validity of the GCS and align with previous research linking corruption to norm-breaking strategies [37] and generalized distrust [46].

## Conclusions

Despite meeting its goals and presenting a measure with robust evidence of validity and reliability for investigating corruption, this research has limitations. A noteworthy limitation concerns the number of participants in Studies 2 and 3 who failed the attention check item. Future studies could adopt alternative data collection strategies or implement additional attention checks to ensure more reliable data and reduce the likelihood of response biases such as careless responding [48], which can compromise the psychometric quality of instruments.

Furthermore, the sample was confined to the Brazilian context, which may restrict the generalizability of the findings to other countries. Thus, future research should also examine whether the scale’s structure is replicable in cultural contexts outside Brazil. Although its development was tailored to the Brazilian setting (i.e., the items were developed to capture context-specific aspects of corruption in Brazil), it is based on a comprehensive theoretical model that incorporates multiple levels of analysis. It is essential to test whether the structure holds in other countries and cultures. As has been widely discussed in psychology - particularly in the last 15 years - the production of psychological knowledge must be contextualized to specific social and cultural characteristics [29, 49]. This principle applies to the *General Corruption Scale*, which should be adapted and validated across contexts to evaluate its psychometric structure and other relevant conceptual parameters.

This study contributes to the literature by providing researchers with a general measure of corruption that does not rely on specific scenarios or subtypes, but instead is grounded in conceptual elements defined by the Analytical Model of Corruption [3]. As such, the GCS can be applied to assess general corruption tendencies in the broader population, including individuals outside of corporate or governmental roles. It thus represents an important tool for self-report research, especially in large-scale studies with diverse samples. Moreover, it may support research and evidence-based discussions aimed at understanding corruption processes and informing the development and evaluation of public policies designed to prevent and address corruption.

## Data Availability

Data and are available in the Open Science Framework at https://osf.io/ew38n/overview? view_only=9c9dbd641cbf442baa90c0a6a41a50f1.

## Abbreviations

GCS: General Corruption Scale
CCI: Control of Corruption Index
CI: Corruption Index
CPI: Corruption Perceptions Index
TPB: Theory of Planned Behavior
AMC: Analytical Model of Corruption
CFA: Confirmatory Factor Analysis
CVC: Content Validity Coefficient
CL: Clarity of Language
PR: Practical Relevance
TR: Theoretical Relevance
TD: Theoretical Dimension
EFA: Exploratory Factor Analysis
RMSR: Root Mean Square Residual
DWLS: Diagonally Weighted Least Squares
CFI: Comparative Fit Index
GFI: Goodness of Fit Index
RMSEA: Root Mean Square Error of Approximation
JS: Jeitinho Simpático
JM: Jeitinho Malandro
